# Paramagnetic States in Oxygen-Doped Boron Nitride Extend Light Harvesting and Photochemistry to the Deep Visible Region

**DOI:** 10.1021/acs.chemmater.2c01646

**Published:** 2023-02-25

**Authors:** Elan D. R. Mistry, Daphné Lubert-Perquel, Irena Nevjestic, Giuseppe Mallia, Pilar Ferrer, Kanak Roy, Georg Held, Tian Tian, Nicholas M. Harrison, Sandrine Heutz, Camille Petit

**Affiliations:** † Institute of Molecular Sciences and Engineering, Department of Chemistry, Imperial College London, Molecular Sciences Research Hub, White City Campus, 82 Wood Lane, London W12 0BZ, United Kingdom; ‡ London Centre for Nanotechnology and Department of Materials, Imperial College London, South Kensington Campus, Prince’s Consort Road, London SW7 2BP, United Kingdom; § Diamond Light Source Ltd., Diamond House, Harwell Science and Innovation Campus, Didcot OX11 0DE, United Kingdom; ∥ Barrer Centre, Department of Chemical Engineering, Imperial College London, South Kensington Campus, Exhibition Road, London SW7 2AZ, United Kingdom

## Abstract

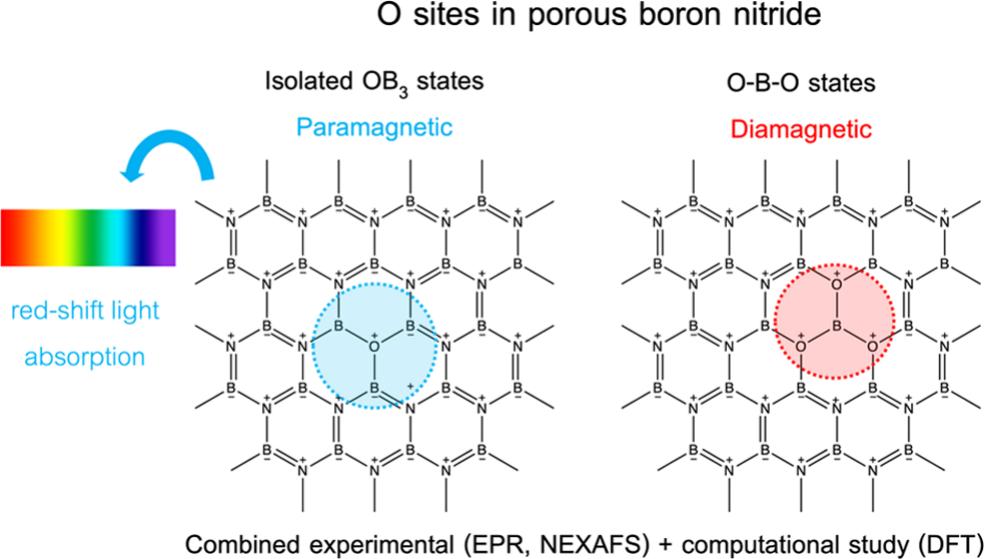

A family of boron nitride (BN)-based photocatalysts for solar fuel syntheses have recently emerged. Studies have shown that oxygen doping, leading to boron oxynitride (BNO), can extend light absorption to the visible range. However, the fundamental question surrounding the origin of enhanced light harvesting and the role of specific chemical states of oxygen in BNO photochemistry remains unanswered. Here, using an integrated experimental and first-principles-based computational approach, we demonstrate that paramagnetic isolated OB_3_ states are paramount to inducing prominent red-shifted light absorption. Conversely, we highlight the diamagnetic nature of O–B–O states, which are shown to cause undesired larger band gaps and impaired photochemistry. This study elucidates the importance of paramagnetism in BNO semiconductors and provides fundamental insight into its photophysics. The work herein paves the way for tailoring of its optoelectronic and photochemical properties for solar fuel synthesis.

## Introduction

The optoelectronic properties of boron nitride (BN) have been increasingly studied to explore the application of the material in fields such as photocatalysis, photovoltaics and electronics, sensing, and quantum emission.^1−7^ One can employ heteroatomic doping to tune the optoelectronic properties of BN with C-doping and, more recently, O-doping, dominating the literature so far.^8−11^ Here, we explore the latter form of doping, during which oxygen atoms substitute nitrogen atoms, leading to the formation of the so-called boron oxynitride (here referred to as BNO). As a result of this doping, BNO exhibits semiconducting and magnetic properties,^9,12−14^ which can be exploited in photocatalysis.^1−4^ In a recent study,^15^ we investigated oxygen doping in BN and presented a route to readily tune and predict the relative oxygen content and optical band gap concomitantly using a design of experiments approach. Detailed statistical analysis revealed a strong inverse relationship between the oxygen content and band gap, which experimentally validated the computational results of Weng et al.^9^ However, the role of different chemical states of oxygen in band gap narrowing in BNO remains unclear, and an understanding of these mechanisms would lead to the design of improved semiconductors.

In the work herein, we delve deeper into the fundamental photochemistry of BNO and aim to shed light on the origins of the visible-range band gaps previously observed. The underlying research question framing this study is: *which chemical state in BNO contributes to red-shifted optical band gaps and enhanced photochemistry?* Our hypothesis is twofold. First, we postulate that paramagnetic, isolated OB_3_ states (*i.e.*, a single N atom is substituted with an O atom without neighboring N atoms substituted) are responsible for lowering the band gap to the deep visible region in BNO. We predict that a higher concentration of paramagnetic, isolated OB_3_ states would lead to enhanced light harvesting. Second, we claim that adjacent OB_3_ centers (*i.e.*, two or more neighboring N atoms are substituted with an O atom) yield diamagnetic O–B–O species (formed by placing OB_3_ centers adjacent to each other) akin to those in boron oxide, which cause undesired blue-shifted band gaps, unlike the paramagnetic, isolated OB_3_ states.

Here, we address our research question and hypotheses using a combined experimental and first-principles-based computational approach. Our experimental characterization was previously conducted on a large BNO sample set, spanning a range of oxygen contents (2–14 atom %) and apparent band gaps (1.50–2.90 eV).^15^ Through a combination of room temperature X-band electron paramagnetic resonance (EPR) spectroscopy and UV–vis diffuse reflectance (DR) spectroscopy, we present an inverse correlation between the magnitude of the paramagnetic OB_3_ signatures and the corresponding apparent band gaps. The experimental trends were substantiated by density functional theory (DFT) simulations of BNO nanosheets: the oxygen dopant introduces a localized state in the band gap of the original undoped boron nitride and significantly red-shifted light-harvesting capability with the presence of multiple paramagnetic OB_3_ states. Using a combination of spectroscopic techniques (X-ray photoelectron spectroscopy (XPS), near-edge X-ray absorption fine structure (NEXAFS), UV–vis DR, and X-band EPR), supported by DFT simulations, we experimentally show that the presence of O–B–O sites from adjacent OB_3_ states leads to diamagnetic states and undesired blue shifts in the absorption spectrum, relative to paramagnetic, isolated OB_3_ states. Our results provide fundamental insight into the photophysics of BNO and show that among the chemical states of oxygen in BNO, paramagnetic, isolated OB_3_ sites appear to have a significant influence on the optoelectronic and photochemical properties of BNO.

## Experimental Section and Theory

### Synthesis of BNO

In a typical synthesis, a reaction mixture (total of 60 mmol) of boric acid (H_3_BO_3_, ACS reagent, 99.0%, Sigma-Aldrich) and hexamethylenetetramine (HMTA) (C_6_H_12_N_4_, molecular biology grade, Sigma-Aldrich) in varying molar ratios (1:2, 2:1, and 5:1) were added to 100 mL of deionized water at 90 °C under rapid stirring to form a boric acid-HMTA complex in solution. The solution was allowed to evaporate overnight until the resulting white powder was collected and subsequently dried for 24 h at 90 °C in a drying oven. The dried material was transferred to an alumina boat crucible (approx. 1.4 g), which was placed in a horizontal tubular furnace. The sample was initially maintained at ambient temperature for 30 min under pure ammonia flow, with the flow rate set to 250 mL min^–1^ to establish an ammonia-rich atmosphere. Caution: ammonia is a toxic gas. A risk analysis must be performed accompanied by the deployment of appropriate safety measures (e.g., ventilation and alarm systems) prior to using this gas. Once this step was complete, the ammonia flow rate was set to a chosen flow rate (50, 150, or 250 mL min^–1^), and the sample was heated from ambient temperature to a set temperature (800, 1000, or 1200 °C), with a ramp rate of 10 °C min^–1^. This steady-state temperature was maintained for 3 h, after which the samples were allowed to naturally cool to approximately 600 °C under the same ammonia flow rate. At this point, the ammonia flow was shut off, and inert argon gas was flowed through at a rate of 100 mL min^–1^ overnight until the furnace had cooled to room temperature. Upon completion of the synthesis, either light brown or yellow powders were obtained, which we refer to as BNO.

### Synthesis of m-BNO

The first step involves the synthesis of monoclinic metaboric acid polymeric precursor ([B_3_H_3_O_6_]*_n_*), using an adapted method of Bertoluzza et al.^16^ Briefly, 3.0 g of boric acid (H_3_BO_3_, ACS reagent, 99.0%, Sigma-Aldrich) was weighed and transferred to a glass reagent bottle. This bottle was placed in a drying oven at 90 °C for 24 h with the lid open to release water and promote dehydration to form orthorhombic metaboric acid. After 24 h, the lid was sealed to prevent hydration, and the oven temperature was increased to 140 °C. This temperature was maintained for a further 24 h to yield monoclinic metaboric acid. The second step involves the reaction of monoclinic metaboric acid with hexamethylenetetramine (HMTA) (C_6_H_12_N_4_, molecular biology grade, Sigma-Aldrich). The synthesis conditions were matched to those for the synthesis of BNO exhibiting the highest OB_3_ intensity. Then, 40 mmol of monoclinic metaboric acid and 20 mmol of HMTA were thoroughly mechanically mixed and transferred to an alumina boat crucible (approx. 1.4 g), which was placed in a horizontal tubular furnace. The sample was initially maintained at ambient temperature for 30 minutes under pure ammonia flow, with the flow rate set to 250 mL min^–1^ to establish an ammonia-rich atmosphere. Caution: ammonia is a toxic gas. A risk analysis must be performed accompanied by the deployment of appropriate safety measures (e.g., ventilation and alarm systems) prior to using this gas. Once the degassing was complete, the ammonia flow rate was set to 50 mL min^–1^, and the sample was heated from ambient temperature to a set temperature of 800 °C, with a ramp rate of 10 °C min^–1^. This steady-state temperature was maintained for 3 h, after which the samples were allowed to naturally cool to approximately 600 °C under the same ammonia flow rate. At this point, the ammonia flow was shut off, and inert argon gas was flowed through at a rate of 100 mL min^–1^ overnight until the furnace had cooled to room temperature. Upon completion of the synthesis, a light yellow/white powder was obtained, which we refer to as m-BNO.

### Electron Paramagnetic Resonance (EPR) Spectroscopy

EPR experiments were carried out using a Bruker Elexsys E500 CW EPR spectrometer operating at X-band frequencies (9–10 GHz/0.3 T), equipped with a Bruker ER4118-X MD5 resonator. All spectra were recorded at room temperature in an air atmosphere in 4mm EPR Suprasil tubes. Spectra were acquired using 0.2 mW of microwave power with field modulation of 100 kHz frequency and 2G modulation amplitude in the detection sequence.

### UV–vis Diffuse Reflectance (UV–vis DR) Spectroscopy

UV–vis DR spectroscopy was conducted using a Shimadzu UV-2600 true optical double beam UV–vis spectrophotometer equipped with an integrating sphere. The integrating sphere has an InGaAs detector with a detection range of 220–1400 nm. Spectral bandwidth was set to 5 nm, and barium sulfate (BaSO_4_) was used as a standard for the baseline corrections. Spectra were treated using the Kubelka–Munk function to eliminate any tailing contribution from the UV–vis DR spectra. The following equation was applied: *F*(*R*) = (1 – *R*)^2^/2*R*, where *R* is the reflectance (%). The apparent band gaps (*E*_G_) were estimated *via* extrapolation of the linear section of the Tauc plot of [*F*(*R*)·*h*v]^1/*n*^ against photon energy (*h*ν). We consider BNO and m-BNO as direct band gap (*n* = 0.5) semiconductors based on the literature.^17^

### XPS Measurements

XPS was employed to determine the relative elemental composition of the samples and the chemical states of the elements. This was conducted using a Thermo Scientific K-α^+^ X-ray Photoelectron Spectrometer equipped with a MXR3 Al Kα monochromated X-ray source (*h*ν = 1486.6 eV). The samples were initially ground and mounted onto an XPS sample holder using a small rectangular piece of conductive carbon tape. The X-ray gun power was set to 72 W (6 mA and 12 kV). Survey scans were acquired using 200 eV pass energy, 0.5 eV step size, and 100 ms (50 ms × 2 scans) dwell times. All of the high-resolution core level spectra (B 1s, N 1s, C 1s, and O 1s) were obtained using a 20 eV pass energy and 0.1 eV step size. Any charging effect in the core level was mitigated using a dual-beam flood gun that uses a combination of low-energy electrons and argon ions.

### NEXAFS Spectroscopy

Near-edge X-ray absorption fine structure (NEXAFS) spectroscopy experiments were carried out on the beamline B07 at the Diamond Light Source Synchrotron, U.K.^18^ Samples were ground as powders and mounted onto carbon tape. We used the 400 lines/mm Pt gratings of the beamline’s plane grating monochromator with an exit slit width of 50 μm, which leads to an energy resolution of 50 and 200 meV at the B and O K-edges, respectively. B and O K-edge spectra were recorded in total electron yield (TEY) mode at room temperature under ultrahigh vacuum (UHV, typically 10^–8^ mbar).

### DFT Simulations

All calculations were performed using the CRYSTAL17 software package, based on the expansion of the crystalline orbitals as a linear combination of a local basis set (BS) consisting of atom-centered Gaussian orbitals with s, p, or d symmetry. The ground-state geometry was optimized for each BNO slab using the global hybrid exchange B3LYP functional to describe electronic exchange and correlation. This functional provides a qualitatively correct correction for electronic self-interaction and thus reproduces the energy, electronic structure, and band gap of self-localizing magnetic states in 2D systems.^19,20^ Listed are the basis sets utilized: boron: B_6-21G*_pople,^21^ nitrogen: N_6-31d1G_gatti_1994,^22^ oxygen: O_8–411_muscat_1999,^23^ and hydrogen: H_5-11G*_dovesi_1984^24^ (see the section basis set in the CRYSTAL webpage: https://www.crystal.unito.it/basis-sets.php). Overall, the model considers a single hBN layer as it is not currently possible to model the exact and complex structure of the actual material. The XRD patterns of the material suggest little crystallinity and therefore a structure closer to amorphous BN (aBN) than hBN. However, in aBN, the BN layers are “arranged” randomly from one another. Hence, considering porous BN as a collection of single BN layers seems like a reasonable approximation. This approach was also adopted in ref (9).

For the calculations, a pruned (99,1454) grid consisting of 99 radial points and 1454 angular points (the XXLGRID option implemented in CRYSTAL17) was adopted due to the ability of converging the integrated charge density to an accuracy of about 10^–6^ electrons per unit cell. A commensurate grid of *k* points in reciprocal space was selected according to the Pack-Monkhorst method (using a shrinking factor 24 for the primitive cell as a reference). The value for the shrinking factor used was dependent on the unit cell size, whereby doubling the cell length both in the *a* and *b* lattice directions halving the grid of *k* points in the reciprocal space. Further, a Gilat net of 48 was used to calculate the density matrix and Fermi energy for higher accuracy. A slab model (periodic along *a* and *b* and not periodic in the perpendicular direction) was used to simulate the surface; in the third direction, the wave function decays to zero at an infinite distance from the surface.

The B3LYP-optimized 2D lattice parameters (*a* and *b*) for the stoichiometric BN slab are *a* = *b* = 2.519 Å and γ = 120°. A nitrogen atom was placed in the unit cell with fractional coordinates (0.0, 0.0, 0.0), and boron was placed with fractional coordinates (0.3333, 0.6666, 0.0). For BNO, a supercell of 2 × 2 was used, and the nitrogen in the origin was replaced with an oxygen atom; within this supercell, a ratio of 4:3:1 of boron, nitrogen, and oxygen, respectively, was used. This concentration of oxygen corresponds to 12.5 atom %. Due to the paramagnetic nature of this material, a spin-polarized calculation was performed.

For the O–B–O system, a supercell of 4 × 4 was used. Oxygen replaced nitrogen atoms with fractional coordinates (0.0, 0.0, 0.0), (0.0, 0.25, 0.0), (−0.5, 0.0, 0.0), and (−0.5, 0.25, 0.0). Within this supercell, a ratio of 4:3:1 of boron, nitrogen, and oxygen, respectively, was adopted again. In this scenario, however, the oxygen atoms were placed such that they were adjacent to each other and not isolated.

For OH-passivated BNO, a supercell of 3 × 3 was defined. The hydroxyl group was placed vertically over a boron, and then the atomic coordinates of the system was optimized, while the hydroxyl group passivating a nitrogen was also placed vertically on a nitrogen atom and then also optimized. Total electron charge and spin densities were calculated for the ideal BNO slab looking above on the *a* and *b* plane and through the *y*,*z* plane and plotted using CRYSPLOT.^25^

The calculations were performed using the spin-unrestricted broken symmetry Kohn–Sham formalism. The magnetization of the cell is equal to the number of OB_3_ sites.

## Results and Discussion

**Figure 1:**
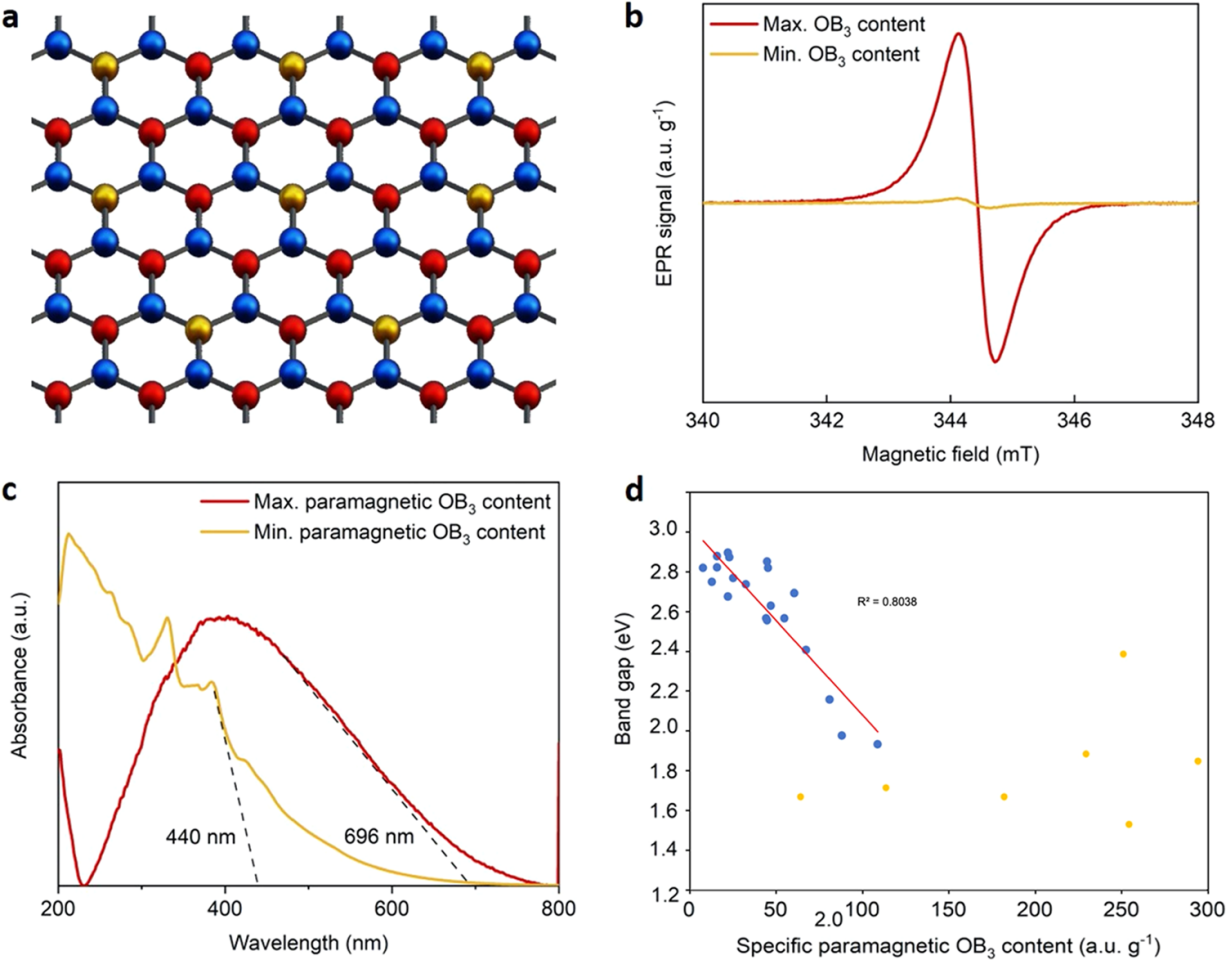
Paramagnetism and apparent band gaps in BNO at *T* = 298 K. (a) Schematic of an “idealized” BNO nanosheet and the isolated OB_3_ states with alternating nitrogen atoms (red) substituted by oxygen atoms (gold) (boron atoms in blue). (b) X-band EPR spectra of the BNO samples exhibiting the highest and lowest specific paramagnetic OB_3_ intensity at room temperature. (c) Absorption spectra for the BNO samples exhibiting the highest and lowest specific paramagnetic OB_3_ intensity. (d) Scatter plot of the experimental apparent band gap and corresponding specific paramagnetic OB_3_ intensity for all BNO samples in this study. Blue data points are those for which we observe a sharp linear decrease of the band gap with increasing specific paramagnetic OB3 intensity from 3.0 to 1.8 eV. For these points, we present linear regression. This set of data is presented in the Supporting Information of ref (15), and we introduce it to support new analyses. Adapted with permission from ref (15). Copyright 2022, Wiley.

We focus first on the characterization of the 27 BNO samples used for this study. Here, we link the EPR patterns to the type of O-containing site and investigate any correlation with the apparent band gap. Based on our hypothesis, a schematic illustration of an “ideal” BNO nanosheet is shown in Figure 1a, with isolated OB_3_ centers formed throughout the lattice by alternate nitrogen atoms being substituted with oxygen. The substitution of O for N in the formation of isolated OB_3_ centers introduces an unpaired electron. This gives rise to a paramagnetic radical signal that can be observed using X-band EPR spectroscopy in ambient conditions.^26^ We normalized the intensity of the radical signal to the sample mass to allow for valid comparison across the sample set. The intensity of the normalized EPR signal is proportional to the number of spins in the system.^27^ Since the spins in BNO originate from radicals in isolated OB3 centers, the intensity of the normalized EPR signal is proportional to the radical content, and hence the number of paramagnetic, isolated OB3 states. We show the BNO samples with the highest and lowest specific paramagnetic OB_3_ content in Figure 1b. We presented the full comparison of the 27 BNO samples in our previous study.^15^ We note that characterization analyses of the samples can be found in this former work (including XRD patterns). While the relative oxygen content of both samples is within 7 atom % of each other (samples 10 and 27 in Table S1), the OB_3_ radical content is around 40 times lower in the BNO sample, exhibiting the minimum EPR signal intensity (Figure 1b and Table S1). This suggests that the doped oxygen atoms are forming diamagnetic states, identified below as O–B–O species. The corresponding absorption spectra for the same BNO samples are shown in Figure 1c. We make here two important notes for contextualization: (i) the relatively high O content of the samples is partly attributed to their amorphous nature, and (ii) we can envision that O atoms will create a distortion in the hBN layers, which might become “visible” for large O contents.

The BNO sample with the highest specific paramagnetic OB_3_ intensity, as measured by EPR, exhibited an absorption edge of 696 nm, corresponding to a deep visible-range apparent band gap of 1.78 eV; this apparent band gap was among the lowest measured among the 27 BNO samples screened in this study (see Table S1 for values). The BNO sample with the lowest paramagnetic OB_3_ intensity exhibited a significantly lower absorption edge of 440 nm, corresponding to an optical band gap of 2.82 eV. This observation suggests that a larger proportion of paramagnetic, isolated OB_3_ states leads to lower apparent band gaps, as visualized by the scatter plot presented in Figure 1d. The scatter plot shows the apparent band gaps of all of the BNO samples synthesized in this study in relation to their specific paramagnetic OB_3_ content. The experimental data was regressed with a least-squares line of best fit (red line in Figure 1d—coefficient of determination *R*^2^ = 0.77), with most points clustered within a 95% confidence interval (gray shaded region in Figure 1d). We note that Figure 1d is included in our earlier contribution^15^ and reused here for comparison with our computational work. However, while the scatter plot has been regressed with a linear fit, close examination appears to suggest two separate regions to the relationship between the specific paramagnetic OB3 intensity and the optical band of BNO. Within the domain of 0–100 au g^–1^, there appears to be a sharp linear decrease of the apparent band gap with increasing specific paramagnetic OB3 intensity from 3.0 to 1.8 eV (visualized in Figure S1). As the specific paramagnetic OB3 intensity increases beyond 100 au g^–1^, implying a greater free radical content, the apparent band gap of BNO appears to plateau at a constant value of 1.5–1.6 eV. This could suggest that beyond a certain concentration of isolated OB3 sites, the increasing presence of free radicals does not decrease the apparent band gap of BNO further but may potentially influence the charge carrier dynamics, carrier mobility, or perhaps conductivity, which would improve photophysical properties for optoelectronics applications. Another explanation could be the influence of varying relative atomic contents of residual carbon from the HMTA precursor in the different samples. Carbon could be incorporated as isolated atoms within the BN structure and/or as “graphene islands” within the hBN layers. While both could exist a priori, we do not see patterns from graphene using the characterization techniques we have employed. In addition, structural features (e.g., layer sizes, porosity) may play a role. Disentangling the contributions of carbon and oxygen as well as structural features on the physical–chemical properties of BN materials remains a challenging task.^28^ Nevertheless, we observe a noticeable inverse relationship between the apparent band gap and specific paramagnetic OB3 content. This supports the notion that the proportion of oxygen chemical states, namely, paramagnetic isolated OB3 sites, may have the most significant influence on the band gap in BNO materials compared to other chemical states of oxygen. This experimentally supports the first part of our hypothesis.

**Figure 2:**
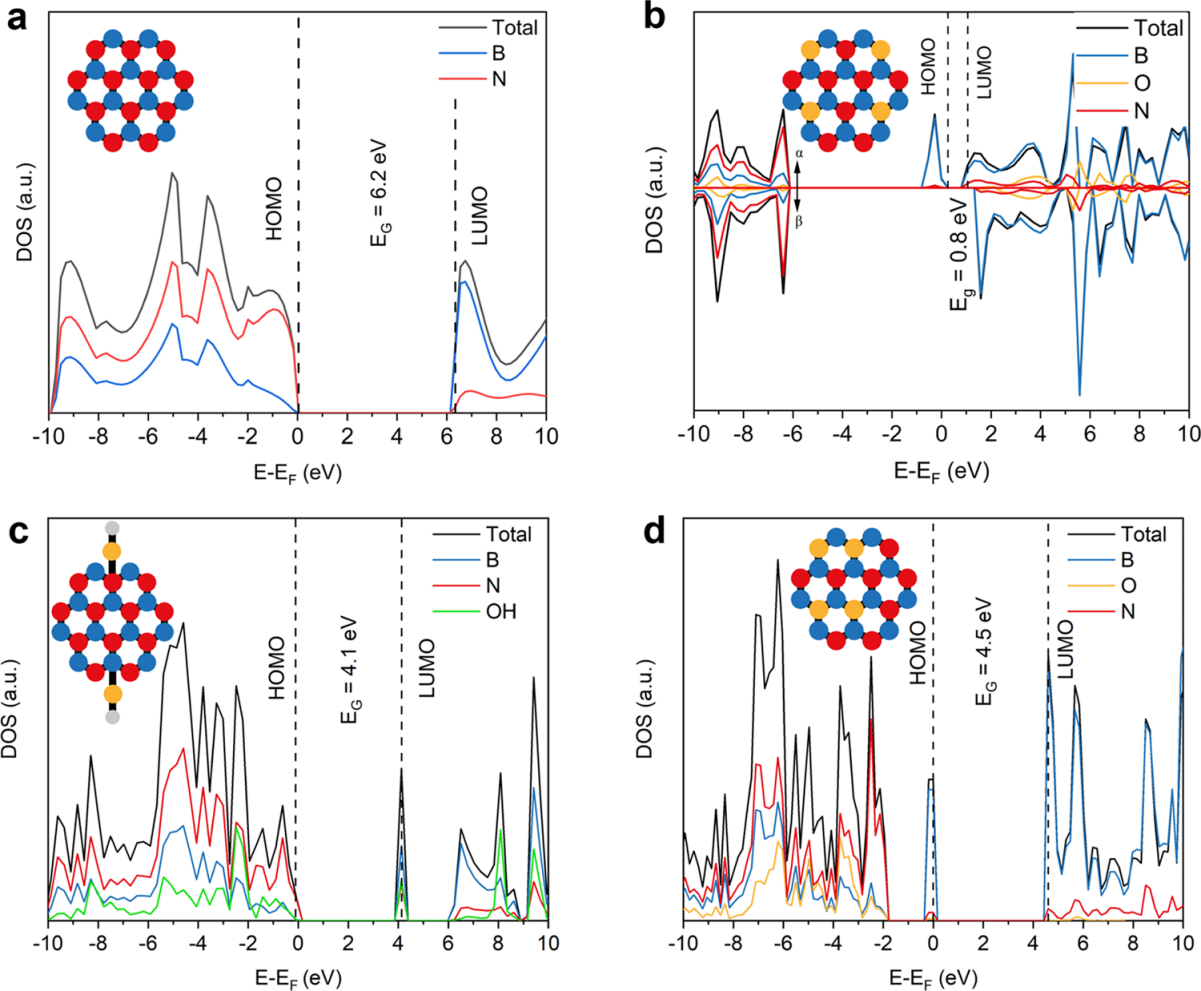
DFT-simulated band structures of different BNO systems. Total- and partial density of states (TDOS and PDOS) plots for: (a) pristine BN sheet with no oxygen atoms. (b) BNO sheet, with all oxygen states as paramagnetic, isolated OB_3_ sites. The contribution from the α and β electrons are labeled. (c) BNO sheet with passivated hydroxyl (−OH) groups and no interior OB_3_ sites. (d) BNO sheet with O–B–O sites and no isolated OB_3_ sites. The inset schematics illustrate the chemical environment of oxygen in each BNO system: boron atoms (blue), nitrogen atoms (red), oxygen atoms (gold), and hydrogen atoms (gray). Note: the acronym HOMO is used in its meaning of the highest occupied molecular orbital in the system under consideration, and this corresponds to a localized state in panels (b) and (d).

**Figure 3:**
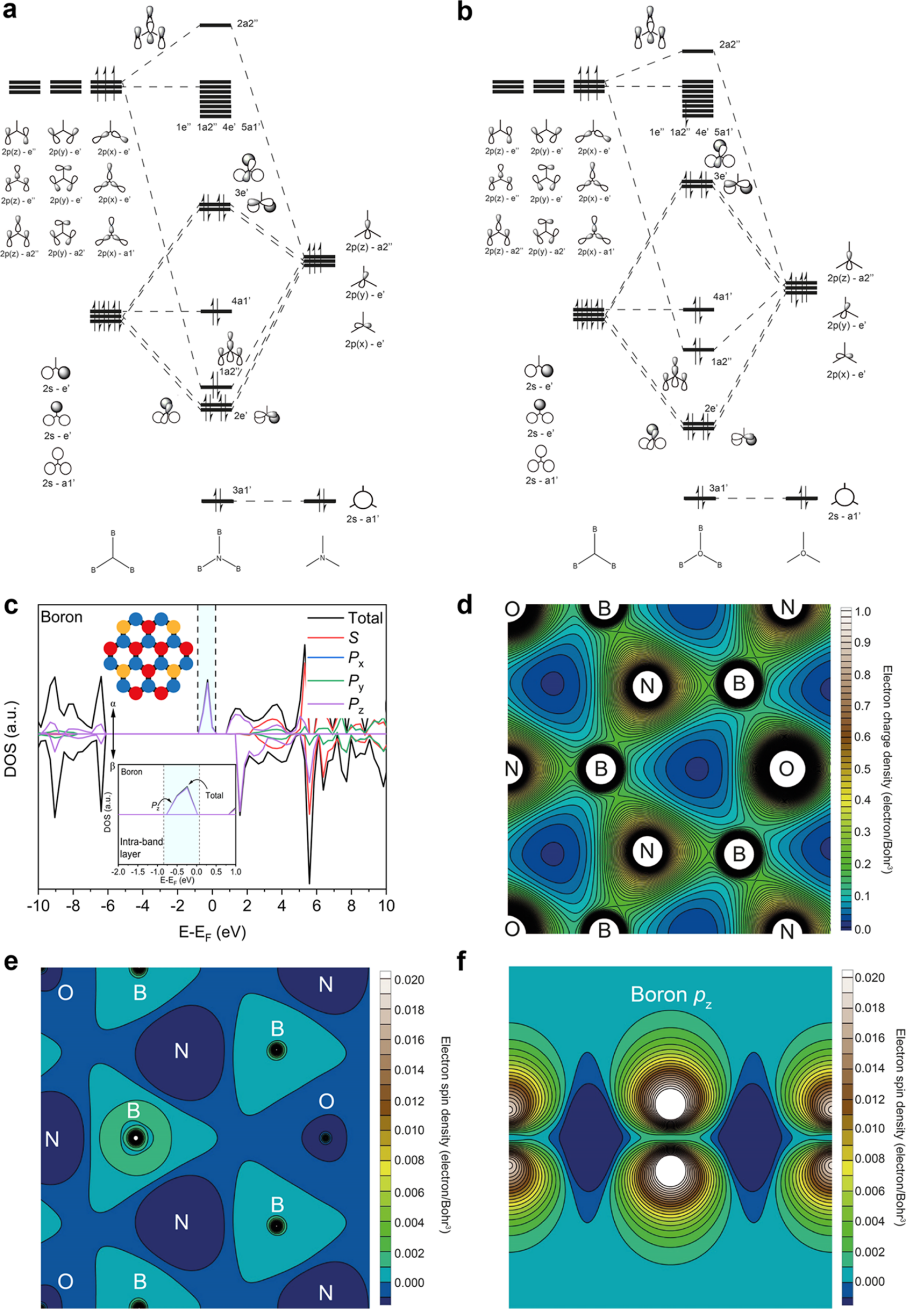
Molecular orbital diagrams for NB_3_ and OB_3_ states, DFT-simulated orbital contributions to dopant state, electron charge- and spin density distribution in BNO. Molecular orbital (MO) diagrams derived from first principles through a linear combination of atomic orbitals (LCAO) for (a), an isolated NB_3_ site and (b), an isolated OB_3_ site. (c) Orbital contributions from boron atoms to TDOS and PDOS in BNO sheet, with all oxygen states as isolated OB_3_ sites. The inset shows the major contribution to the localized state originating from the boron *p*_z_ orbitals. The α and β electrons states are labeled. (d) Distribution of electron charge density for the BNO system shown in Figure 2b, where all oxygen atoms are paramagnetic, isolated OB_3_ states. Distribution of electron spin density for BNO system in Figure 2b, where all oxygen atoms are paramagnetic, isolated OB_3_ states, in (e) *x*–*y* plane (*i.e*., bird’s eye view) and (f), *y*–*z* plane (*i.e*., looking above and below the plane of the BNO lattice). Both the electron charge density and spin density are localized and concentrated about the boron atoms in the paramagnetic isolated OB_3_ states, confirming the radical position in a boron orbital, as predicted by the MO diagram in Figure 3b.

Next, we use Figure 1d to compare the experimental trends between the specific paramagnetic OB_3_ content and band gap with the first-principles DFT calculations in Figures 2 and 3. The observed trends have been explored using DFT calculations (in particular, the global hybrid exchange functional B3LYP as implemented in the periodic quantum-mechanical code CRYSTAL17^29−32^). The calculations provide a fundamental insight into the photochemistry of the oxygen chemical states in BNO. The DFT-simulated total- and partial density of states (TDOS and PDOS) plots for BNO systems with different chemical environments of oxygen are presented in Figure 2a–d. The insets in Figure 2a–d illustrate the chemical bonding nature of oxygen in each system. We first consider the widely studied pristine BN nanosheet with no oxygen atoms to (i) validate the DFT model and (ii) use it as a reference to identify the influence of introducing oxygen to the system. The simulations revealed a large band gap of 6.2 eV (Figure 2a), which agrees with that reported in the literature.^9,10^Figure 2b depicts the BNO system, where every N-substituted oxygen atom forms a paramagnetic, isolated OB_3_ state without the formation of O–B–O sites (see the inset of Figure 2b). We replaced approximately 25% of the nitrogen atoms in the pristine BN sheet (Figure 2a) with oxygen atoms. Due to the paramagnetic nature of this BNO material, we adopted a spin-unrestricted broken symmetry Kohn–Sham formalism. In Figure 2b, positive and negative values for the DOS axis correspond to spin-up (α) and spin-down (β) electrons, respectively.

We observe a distinct decrease in the band gap from 6.2 to 0.8 eV through the addition of multiple paramagnetic, isolated OB_3_ states. The TDOS plot shows the formation of a localized state in BNO through the introduction of paramagnetic, isolated OB_3_ states at ∼0 eV relative to the Fermi level (Figure 2b). Further, the HOMO and the LUMO shift to higher and lower energies, respectively, in comparison to the pristine BN system. As the new HOMO in the multiple paramagnetic OB_3_ system is the localized state of the dopant, there is a smaller energy barrier for the electrons to overcome to be photoexcited to the LUMO, thus increasing the light-harvesting capability. Figure 2b also shows that the majority of the orbital contribution to the DOS in the localized state arises from a boron atom bonded to the central oxygen atom in paramagnetic, isolated OB_3_ states (blue curve in PDOS plots in Figure 2b). The PDOS in Figure 2b therefore suggests that the radical in the paramagnetic, isolated OB_3_ state is located within a boron orbital, which we study further below. We next investigated the influence of hydroxyl (−OH) groups as another chemical state of oxygen on the band gap of BNO (Figure 2c). To do so, we passivated the BNO sheet with −OH groups (−OH adsorption) and did not include any interior substituted OB_3_ sites. The latter was omitted from the simulation to allow us to solely ascertain the contribution of −OH groups on band gap narrowing in BNO. After hydroxylation, we observed that the band gap decreased to 4.1 eV (Figure 2c), which is still within the UV range. We next modeled a BNO system with O–B–O sites (Figure 2d) and compared this to the BNO system with multiple isolated OB_3_ states in Figure 2b. The simulated model with O–B–O sites has the same relative oxygen content as that with multiple isolated OB_3_ states (Figure 2b). The underlying difference is that the OB_3_ centers are placed adjacent to each other to form O–B–O sites. Compared to the BNO system with solely paramagnetic isolated OB_3_ sites (Figure 2b), we observe a larger, blue-shifted band gap of 4.5 eV in the simulated O–B–O system. This supports our hypothesis for this study. We postulate that the blue shift within the simulations, when transitioning from isolated OB_3_ states to O–B–O sites, arises from the chemical bonding associated with the formation of the latter.

As shown above, the radical, derived from the paramagnetic, isolated OB_3_ state, occupies a boron orbital. We postulate that the formation of an O–B–O site from adjacent OB_3_ sites results in two radicals pairing up anti-aligned, which would result in a net S = 0, EPR silent spin state. This means the quantum spin state of the system transitions from a doublet to a singlet, explaining the diamagnetic nature of the O–B–O species. Further simulations revealed that the major contribution to the dopant layer in the O–B–O system (Figure 2d) is from boron s orbitals with small additional contributions from the p_x_ and p_z_ orbitals (Figure S2). This shows that the paired radicals, now on the bridging boron atom, likely predominantly participate in bonding as opposed to conjugation throughout the BNO system. This lack of conjugation could explain the larger band gaps. Therefore, the combined DFT results from Figures 2a–d and S2 confirm that paramagnetic isolated OB_3_ sites are a unique chemical state of oxygen in BNO that leads to red-shifted band gaps to the deep visible region.

In Figure 2b, we observed that the major contribution to the localized state, arising from paramagnetic isolated OB_3_ states, is from a boron atom neighboring the oxygen atom. This suggests that the radical is occupying a boron orbital. We explore this further by constructing molecular orbital (MO) diagrams from first principles, using linear combination of atomic orbitals (LCAO), for an isolated OB_3_ and NB_3_ state (Figure 3a,b, respectively). The MO diagrams provide fundamental insight into the bonding nature of the paramagnetic OB_3_ states and the origins of band gap narrowing. We constructed the MO diagrams through reasoning the orbital symmetries. The spatial and energetic overlaps are based on educated guesses. Therefore, exact orbital energy values are not given. Additionally, although there may be some orbitals within range for spatial and energetic overlap to occur, e.g., boron 2p(*y*) e′ and oxygen 2p(*y*) e′, they are assumed to be weakly interacting in comparison to the boron 2s e′ interaction and the oxygen 2p(*y*) e′, and so they are ignored. Examining the results, NB_3_ (Figure 3a) exhibits a singlet spin state, and the degenerate HOMO occupies an antibonding orbital that possesses an e′ symmetry. This is in contrast to the HOMO in the OB_3_ center (Figure 3b), which is a nonbonding boron 2p orbital, exhibiting a doublet spin state. The key messages from the MO diagrams, derived from first-principles-based linear combination of atomic orbitals (LCAO), are: (i) the formation of an isolated OB_3_ state results in the HOMO being shifted to higher energies, which reduces the HOMO-LUMO gap, and (ii) the radical from isolated OB_3_ states occupies a nonbonding boron 2p orbital, which further supports the DFT results in Figure 2. The location of the radical in a nonbonding boron 2p orbital (Figure 3b) is confirmed through the PDOS plot in Figure 3c, illustrating that the primary contribution from boron to the localized stated in Figure 2b is from the boron p_z_ orbitals.

To further confirm the location of the radical, we plotted the distribution of electron charge- and spin density in the BNO system in Figure 2b, where all oxygen atoms are paramagnetic isolated OB_3_ sites. The electron charge density is concentrated about the boron atom (Figure 3d). In contrast, the electron charge density surrounding N and O atoms is more diffuse further from the center of the atoms (see Figure S3 for further details). Equally, the spin density is localized about the boron atom, as seen in Figure 3e. Figure 3e represents a bird’s eye view of the spin density in the *x*–*y* plane, such that one is looking at the BNO sheet from above. To elucidate the location of the spins, we examined the spin density in the *y*–*z* plane (Figure 3f), which illustrates the spin density localized above and below the plane of the BNO sheet in a boron p_z_ orbital. By combining the results from the electron charge- and spin density (Figure 3d–f) with the DOS plots and MO diagrams (Figures 2b and 3a,b, respectively), we conclude that the radical from paramagnetic, isolated OB3 sites occupies a boron 2pz orbital. As the orientation of the radical-occupied boron 2pz orbitals lies out of the plane, it is theorized that these orbitals delocalize across the BNO sheet through π conjugation with adjacently aligned boron 2pz orbitals. This extended π conjugation in BNO systems with multiple paramagnetic, isolated OB3 sites would result in band gap narrowing. Overall, the experimental and DFT results in Figures 1–3 show that paramagnetic OB_3_ states red-shift light harvesting in BNO to the deep visible region, confirming the first part of our hypothesis.

We now turn our attention to the second part of our hypothesis surrounding the effect of O–B–O states. To compare the behavior of OB_3_ and O–B–O sites, we developed a two-step synthesis route to promote the formation of O–B–O sites over isolated OB_3_ states in a control BNO sample. Whereas BNO samples exhibiting the highest specific paramagnetic OB_3_ intensity use boric acid (H_3_BO_3_) as the precursor, the control was synthesized using monoclinic metaboric acid ([B_3_H_3_O_6_]*_n_*), a polymeric boron precursor with an abundance of O–B–O species. All other synthesis conditions were kept the same. The control sample is labeled and referred to as m-BNO. The formation of monoclinic metaboric acid was confirmed through Fourier transform infrared spectroscopy (FT-IR), in agreement with the literature^16^ (see Figure S7). Using X-ray photoelectron spectroscopy (XPS), the relative oxygen contents and atomic compositions of BNO and m-BNO were found to be virtually identical, with values of 10.6 and 10.5 atom %, respectively (see Table S2 for values). Importantly, the same oxygen content between the two samples allows us to ascribe variations in the magnetic and optoelectronic properties to the chemical states of oxygen.

**Figure 4:**
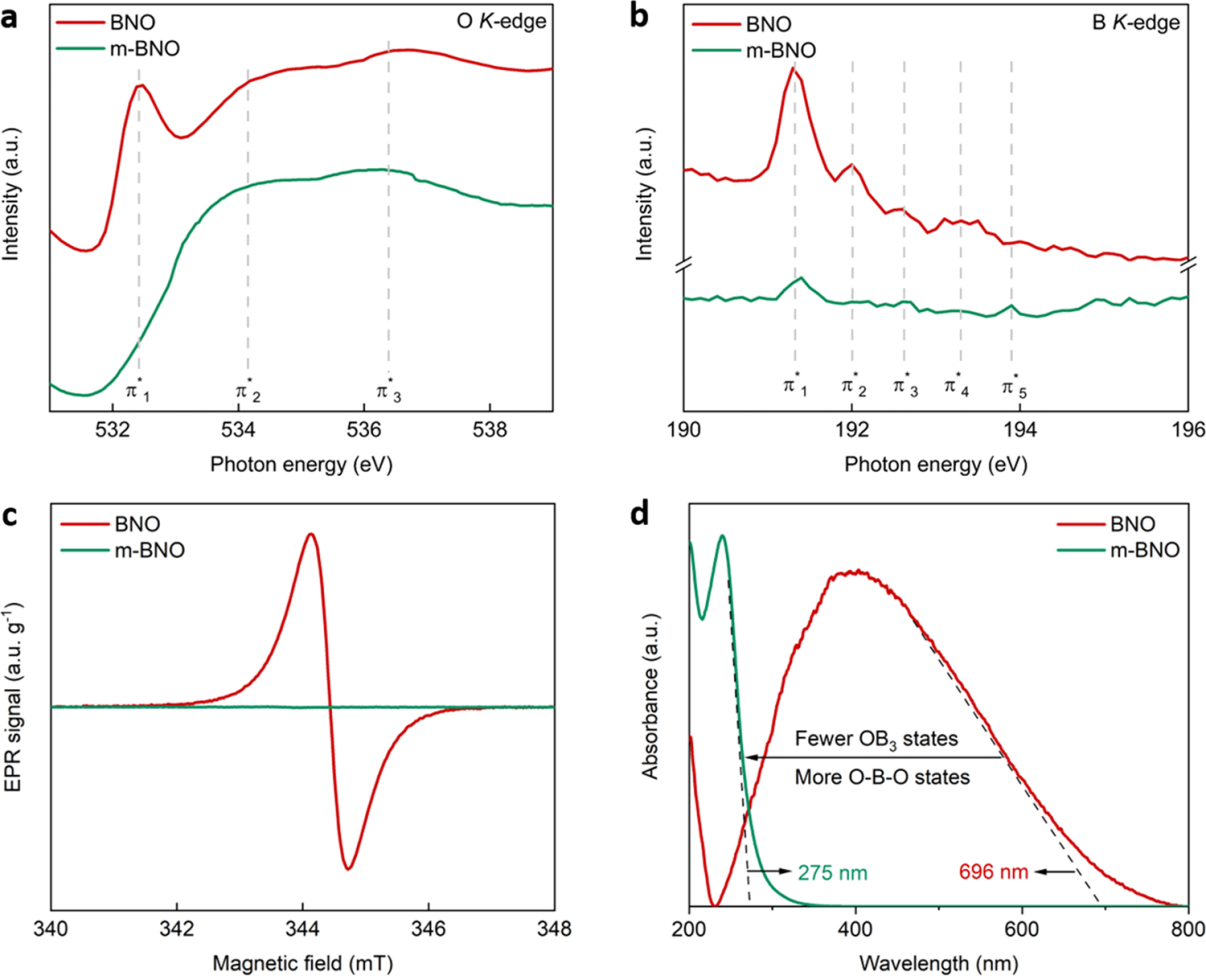
Chemical, magnetic, and optoelectronic properties arising from OB_3_ and O–B–O states at *T* = 298 K. (a) O-K-edge and (b) B-K-edge NEXAFS spectra in BNO and m-BNO. The inset schematics depict the chemical states of oxygen in relation to the corresponding π* transition peaks: boron atoms (blue), nitrogen atoms (red), and oxygen atoms (gold). (c) X-band EPR spectra comparing the specific paramagnetic OB_3_ intensities of BNO and m-BNO. (d) Absorption spectra for BNO and m-BNO, highlighting the diminished light-harvesting capability caused by the absence of OB_3_ states and the formation of O–B–O sites.

To identify the chemical states in BNO and m-BNO, we recorded O and B *K* near-edge X-ray absorption fine structure (NEXAFS) spectra (Figure 4a,b) and compared these with band structure calculations (Figures 2 and S8). The near-edge absorption spectra represent electronic excitations from O 1s or B 1s orbitals into unoccupied states above the Fermi energy. Dipole selection rules only allow transitions into p-like states overlapping with the respective atoms, which are depicted in Figure S8. Neither orbital overlap nor lifetime broadening of excited states is considered in the calculations; therefore, the height and width of the calculated projected DOS do not necessarily correspond to that of the NEXAFS spectra. We focus first on the O K-edge spectra, where we observe a distinct broad peak at 532.4 eV (π_1_*) in the O K-edge spectrum for BNO (Figure 4a), attributed to the π* transitions in the BN_2_O state, where one nitrogen atom is substituted by one oxygen atom,^9,12,33^ forming an isolated OB_3_ coordination. We associate this peak with the p_z_ states between 1.3 and 3.1 eV in the BNO O-projected DOS of Figure S8. This π_1_* BN_2_O peak is absent in the O K-edge spectrum of m-BNO, indicating the absence of isolated OB_3_ centers. The broad features (π_2_* at 534.1 eV and π_3_* at 536.4 eV), which are observed above the absorption edge (around 533 eV) are associated with transitions into the p states above 4 eV, which dominate the DOS plots of both BNO and m-BNO in Figure S8. Notably, in the O K-edge spectra of BNO or m-BNO (Figure 4a), we do not observe a peak at 538 eV (π_4_*), which would correspond to π* (B–O) transitions in boron oxide (B_2_O_3_).^12,34^ This confirms that both BNO and m-BNO remain BN materials despite oxygen doping, and we have not formed an oxide.

We now turn our attention to the B *K*-spectra. In the DOS calculations shown in Figure S8, the unoccupied p states have nonzero DOS above 0.8 eV for BNO and above 4.3 eV for m-BNO. We would therefore expect the first resonance observed for m-BNO to appear at photon energies approximately 3.5 eV higher than for BNO. For the latter, we observe distinctive peaks at 191.6 eV (π_1_*), 192.0 eV (π_2_*), 192.6 eV (π_3_*), and 193.2 eV (π_4_*), which can be mapped onto the features in the B-projected pDOS for BNO between 0.8 and 4.5 eV (Figure S8). π_1_* is associated with the π* transitions in the BN_3_ state in BNO and m-BNO, confirming the formation of a BN material (Figure 4b).^35,36^ This is supported by the high-resolution B 1s and N 1s core level XPS spectra for BNO and m-BNO (see Figures S5 and S6). π_2_*, corresponds to π* transitions in the BN_2_O state, leading to isolated OB_3_ centers.^35,36^

The B-K-edge spectrum of m-BNO spectrum shows a new peak at 193.9 eV (π_5_*), i.e., 2.6 eV above the lowest energy peak seen for BNO and in reasonably good agreement with the shift of 3.5 eV expected from the calculations. Additional features at higher photon energies are assigned to the pDOS states of m-BNO above 4.3 eV. Weak signals of π_1_*, π_2_*, π_3_*, and π_4_* can also be seen in these spectra, highlighting the fact that the layer is not pure. The NEXAFS spectra in Figure 4a,b thus confirm: (i) isolated OB_3_ centers are the dominant chemical state of oxygen in BNO, and (ii) O–B–O species are the dominant state in m-BNO.

The disparity of chemical states in BNO and m-BNO is reflected in the contrasting magnetic signatures, as measured by EPR spectroscopy (Figure 4c). BNO exhibits a radical signal proportional to the specific paramagnetic OB_3_ content, while no such signal was detected for m-BNO. This confirms the diamagnetic nature of O–B–O sites and m-BNO. The paramagnetic and diamagnetic states of BNO and m-BNO, respectively, bring about distinctly different optoelectronic properties, as shown by the absorption spectra in Figure 4d.

BNO, exhibiting the highest paramagnetic OB_3_ intensity, displays a deep visible range apparent band gap of 1.78 eV. However, the increased presence of diamagnetic O–B–O states and absence of paramagnetic, isolated OB_3_ centers in m-BNO leads to a significant blue shift in the apparent band gap to 4.51 eV. The key conclusion from Figure 4a–d is that a higher proportion of diamagnetic O–B–O species results in a significantly larger apparent band gap when compared to the apparent band gap narrowing achieved with multiple paramagnetic, isolated OB3 sites. Naturally, this severely restricts the light-harvesting capability of the material, which in turn can impair its photochemistry.

## Conclusions

To conclude, we have demonstrated through a combined experimental and first-principles-based computational approach that paramagnetic isolated OB_3_ centers in BNO are chemical states that red-shift light absorption and photochemistry to the deep visible region. This extended π conjugation in BNO systems with multiple paramagnetic, isolated OB3 sites seems to result in band gap narrowing. Employing room temperature X-band EPR spectroscopy and UV–vis diffuse reflectance (DR) spectroscopy, we presented an inverse correlation between the magnitude of the paramagnetic OB_3_ signatures and the corresponding apparent band gaps over a set of 27 BNO samples. The DFT simulations provided additional evidence that paramagnetic, isolated OB_3_ states appear to be the sole chemical state of oxygen that can facilitate light harvesting in the deep visible region in BNO. We developed a first-principles theoretical framework using molecular orbital (MO) theory and linear combination of atomic orbitals (LCAO) to gain fundamental insight into the chemical bonding and location of the radical in the paramagnetic, isolated OB_3_ sites. The MO diagrams predicted that the radical occupies a nonbonding 2p orbital on the boron atom. This agrees with DFT simulations, illustrating the electron charge- and spin density to be localized about the boron atom in a p_z_ orbital in the paramagnetic OB_3_ sites. Thus, we conclude that the radical from paramagnetic, isolated OB3 sites occupies a nonbonding boron 2pz orbital. A synthesis route was developed to produce m-BNO, a control sample, with the same relative oxygen content as BNO but O–B–O sites instead of isolated OB_3_ sites. Using NEXAFS spectroscopy, we confirmed the presence of paramagnetic isolated OB_3_ sites in BNO and the absence of such states in m-BNO, which only appeared to exhibit diamagnetic O–B–O sites. The contrasting chemical and magnetic states in BNO and m-BNO yielded significant red- and blue-shifted light-harvesting behavior, respectively. The study herein not only provides valuable insight into the photochemistry of BNO at the fundamental level but also brings to light the importance of paramagnetism in tailoring and optimizing the optoelectronic and photochemical properties of BNO.

## Supporting Information Available

Experimental details of the samples in this study (incl. data extracted from XPS, EPR, FT-IR and UV–vis analyses) along with supporting DFT results (PDF)
Additional files include the final Cartesian coordinates and lattice parameters for the ideal OB_3_ and BN structures (ZIP)
