# Supplementary material for: Paramagnetic States in Oxygen-Doped Boron Nitride Extend Light Harvesting and Photochemistry to the Deep Visible Region
Source: Chem Mater. 2023 Feb 25;35(5):1858–67. doi: 10.1021/acs.chemmater.2c01646 (PMC10018733; doi:10.1021/acs.chemmater.2c01646)
Supplement: Supplementary file 1 — cm2c01646_si_001.pdf [file cm2c01646_si_001.pdf]

Supplementary information

Paramagnetic states in oxygen-doped boron nitride extend light harvesting and photochemistry to deep visible region

Elan D.R. Mistry[a, ¶], Daphné Lubert-Perquel[b,†], Irena Nevjestic[b], Giuseppe Mallia[a], Pilar Ferrer[c], Kanak Roy[c,‡], Georg Held[c], Tian Tian[d], Nicholas M. Harrison[a], Sandrine Heutz[b] and Camille Petit[d*]

[a]Institute of Molecular Sciences and Engineering, Department of Chemistry, Imperial College London, Molecular Sciences Research Hub, White City Campus, 82 Wood Lane, London W12 0BZ, United Kingdom

[b]London Centre for Nanotechnology and Department of Materials, Imperial College London, South Kensington Campus, Prince's Consort Road, London SW7 2BP, United Kingdom

[c] Diamond Light Source Ltd., Diamond House, Harwell Science and Innovation Campus, Didcot OX11 0DE, United Kingdom

[d]Barrer Centre, Department of Chemical Engineering, Imperial College London, South Kensington Campus, Exhibition Road, London SW7 2AZ, United Kingdom

[¶] Current address: Department of Chemical Engineering, University College London, London, WC1E 7JE, United Kingdom

[†]Current address: National Renewable Energy Laboratory, 15013 Denver West Parkway Golden, CO 80401, Unites States

‡Current address: Department of Chemistry, Banaras Hindu University, Varanasi, Uttar Pradesh 221005, India

*Corresponding author: E-mail: camille.petit@imperial.ac.uk; Phone: +44 (0)20 7594 3182 (C. Petit)

Table S1 I Relative atomic compositions obtained through XPS analysis and summary of the specific paramagnetic OB_3 intensities and band gaps of all the BNO samples investigated in this study. The data are taken from our previous study reported here: [1].

Sample	Ratio (-)	Temp. (ºC)	Flowrate (mL min^{-1})	B (at. %)	C (at. %)	N (at. %)	O (at. %)	Relative oxygen content (at. %)	Specific OB_3 intensity (a.u. g^{-1})	Band gap (eV)
1	1:2	800	50	43.07	13.52	32.79	10.62	10.62	108.8	1.94
2	1:2	800	150	46.63	7.84	37.55	7.98	7.98	87.7	1.98
3	1:2	800	250	47.73	6.20	38.55	7.50	7.50	81.0	2.16
4	1:2	1000	50	48.70	5.62	38.67	7.01	7.01	60.1	2.69
5	1:2	1000	150	50.58	3.94	42.01	3.49	3.49	15.9	2.88
6	1:2	1000	250	50.57	4.10	42.40	2.93	2.93	44.6	2.85
7	1:2	1200	50	51.06	3.83	42.18	2.92	2.92	45.1	2.82
8	1:2	1200	150	49.50	6.37	40.69	3.44	3.44	21.7	2.89
9	1:2	1200	250	50.70	3.89	42.02	3.39	3.39	22.8	2.87
10	2:1	800	50	44.67	10.24	34.50	10.59	10.59	294.7	1.78
11	2:1	800	150	44.08	11.05	34.82	10.05	10.05	229.6	1.81
12	2:1	800	250	46.65	7.30	35.74	10.31	10.31	255.1	1.50
13	2:1	1000	50	47.28	7.95	37.40	7.38	7.38	251.6	2.26
14	2:1	1000	150	50.97	3.21	42.23	3.60	3.60	43.5	2.57
15	2:1	1000	250	51.14	2.90	42.91	3.05	3.05	44.5	2.56
16	2:1	1200	50	51.90	2.46	42.75	2.89	2.89	46.6	2.63
17	2:1	1200	150	52.36	1.98	43.52	2.13	2.13	24.7	2.77
18	2:1	1200	250	51.92	2.34	43.47	2.27	2.27	32.3	2.74
19	5:1	800	50	47.11	5.04	35.19	12.67	12.67	113.8	1.66
20	5:1	800	150	42.68	12.72	30.83	13.77	13.77	63.9	1.62
21	5:1	800	250	47.37	5.83	37.85	8.96	8.96	182.3	1.62
22	5:1	1000	50	51.41	2.54	41.80	4.27	4.27	66.8	2.41
23	5:1	1000	150	51.59	2.30	43.19	2.93	2.93	21.8	2.68
24	5:1	1000	250	51.51	2.18	42.15	4.16	4.16	55.0	2.57
25	5:1	1200	50	52.03	2.30	42.85	2.82	2.82	12.8	2.75
26	5:1	1200	150	52.29	1.98	43.57	2.15	2.15	15.7	2.82
27	5:1	1200	250	52.27	2.32	42.76	2.64	2.64	7.4	2.82

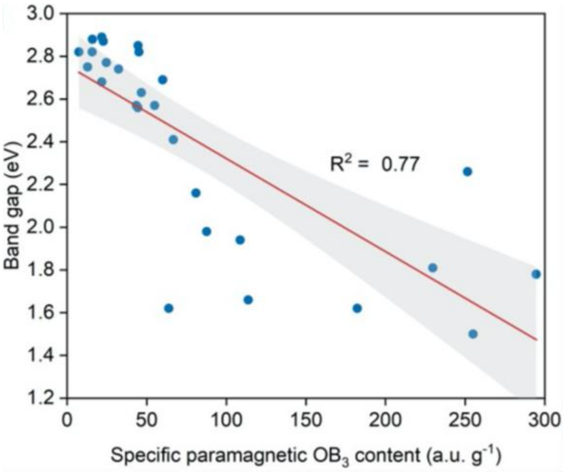

Fig. S1 I Scatter plot of the experimental apparent band gap and corresponding specific paramagnetic OB_3 intensity for all BNO samples in this study, with linear least squares regression (red line) and a 95% confidence region (grey shaded region). This set of data is presented in the Supporting Information of Ref [15] and we introduce it to support new analyses.

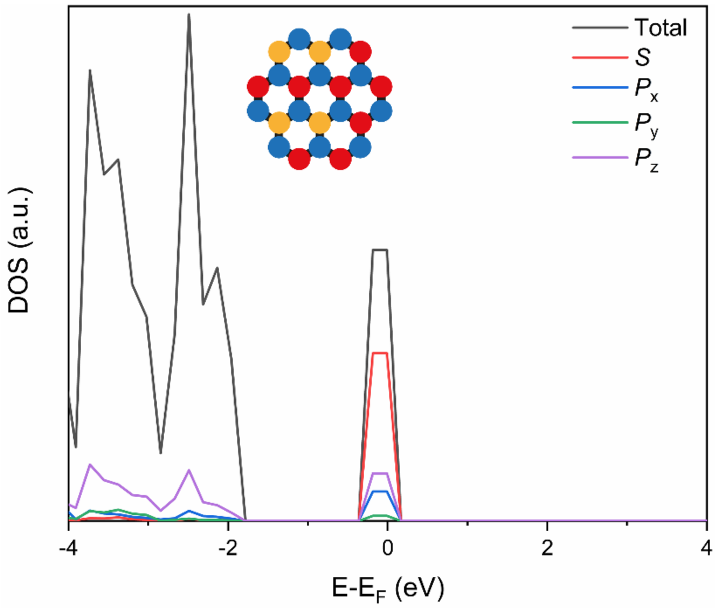

Fig. S2 I Orbital contributions from boron atoms to TDOS and PDOS in BNO sheet with all oxygen atoms as O-B-O states. The major contribution to the intra-band dopant layer in this BNO system is originating from the boron *S* orbitals.

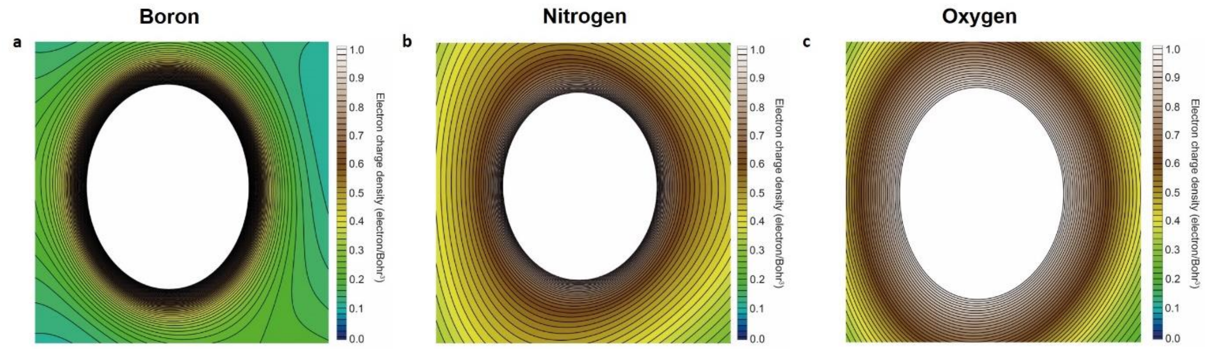

Fig. S3 I Zoomed in distribution of total electron charge density around **a**, boron, **b,** nitrogen and **c**, oxygen atoms for BNO system in Fig. 2b, where all oxygen atoms are paramagnetic isolated OB_3 states. The distribution of total electron charge density surrounding each atom is taken at approximately the same magnification for comparison.

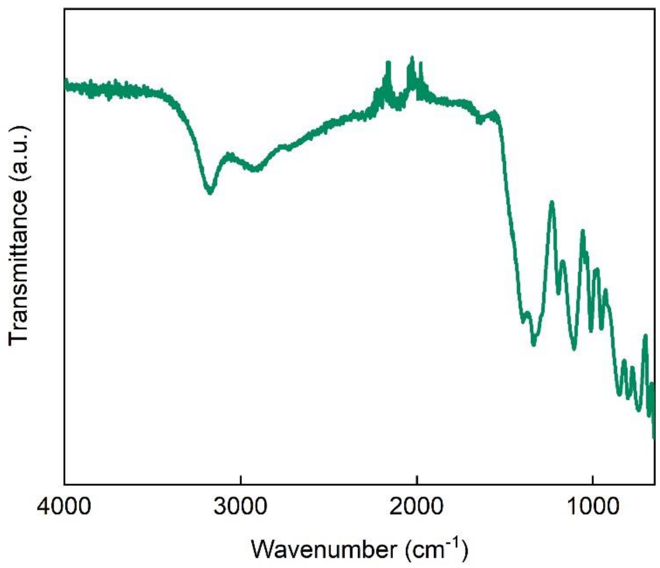

Fig. S4 I FT-IR spectrum of monoclinic metaboric acid ($[B_3H_3O_6]_n$), which is concordant with literature [2].

Table S2 I Summary of the relative elemental compositions of BNO and m-BNO as obtained from XPS measurements.

Sample	B (at. %)	C (at. %)	N (at. %)	O (at. %)
**BNO**	44.67	10.24	34.51	10.59
**m-BNO**	48.21	4.81	36.50	10.48

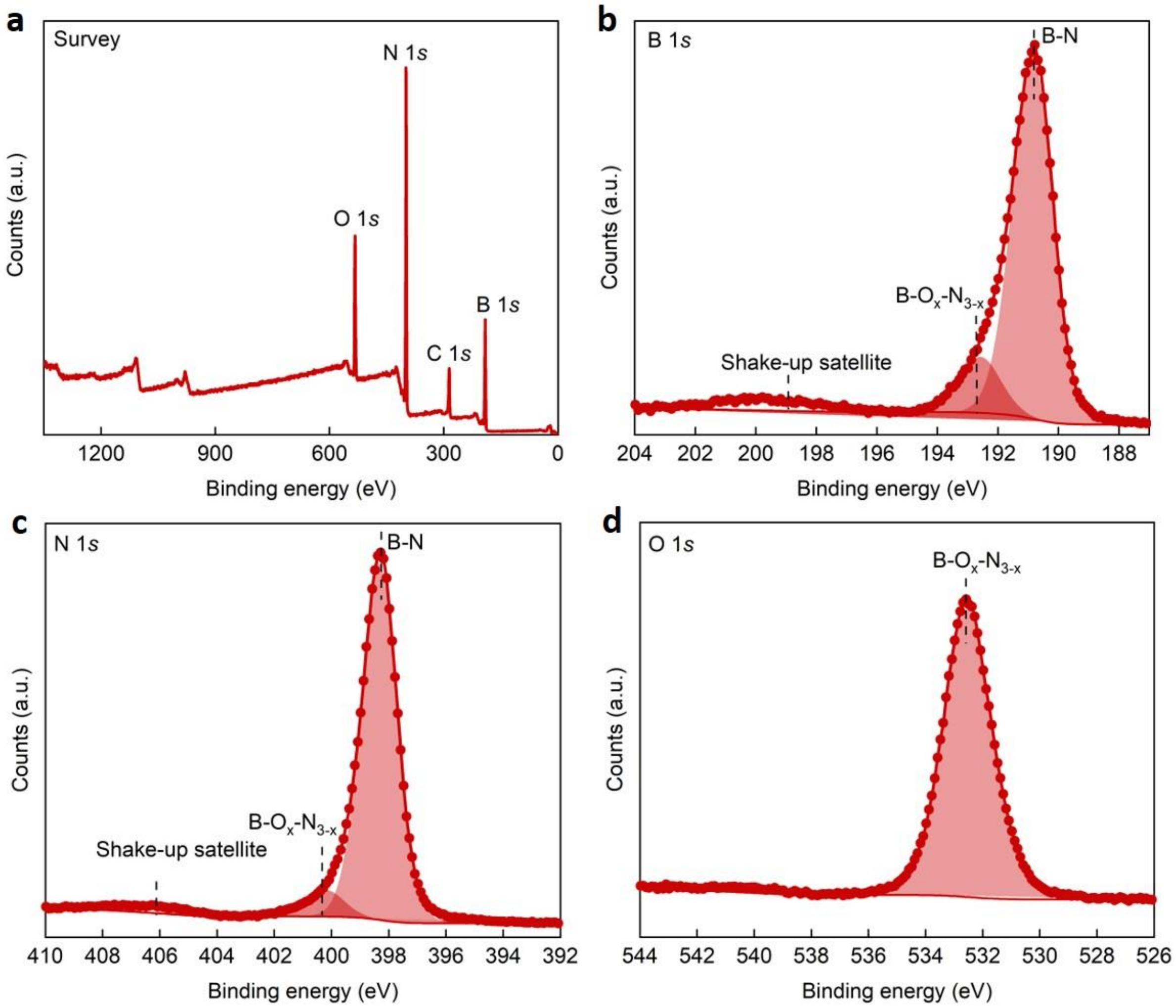

Fig. S5 I Chemical composition and states in BNO. Survey spectrum and high resolution XPS core level spectra for B 1*s*, N 1*s* and O 1*s* with the key characteristic peaks and corresponding binding energies highlighted. Measurements done on Sample 10 (Table 1).

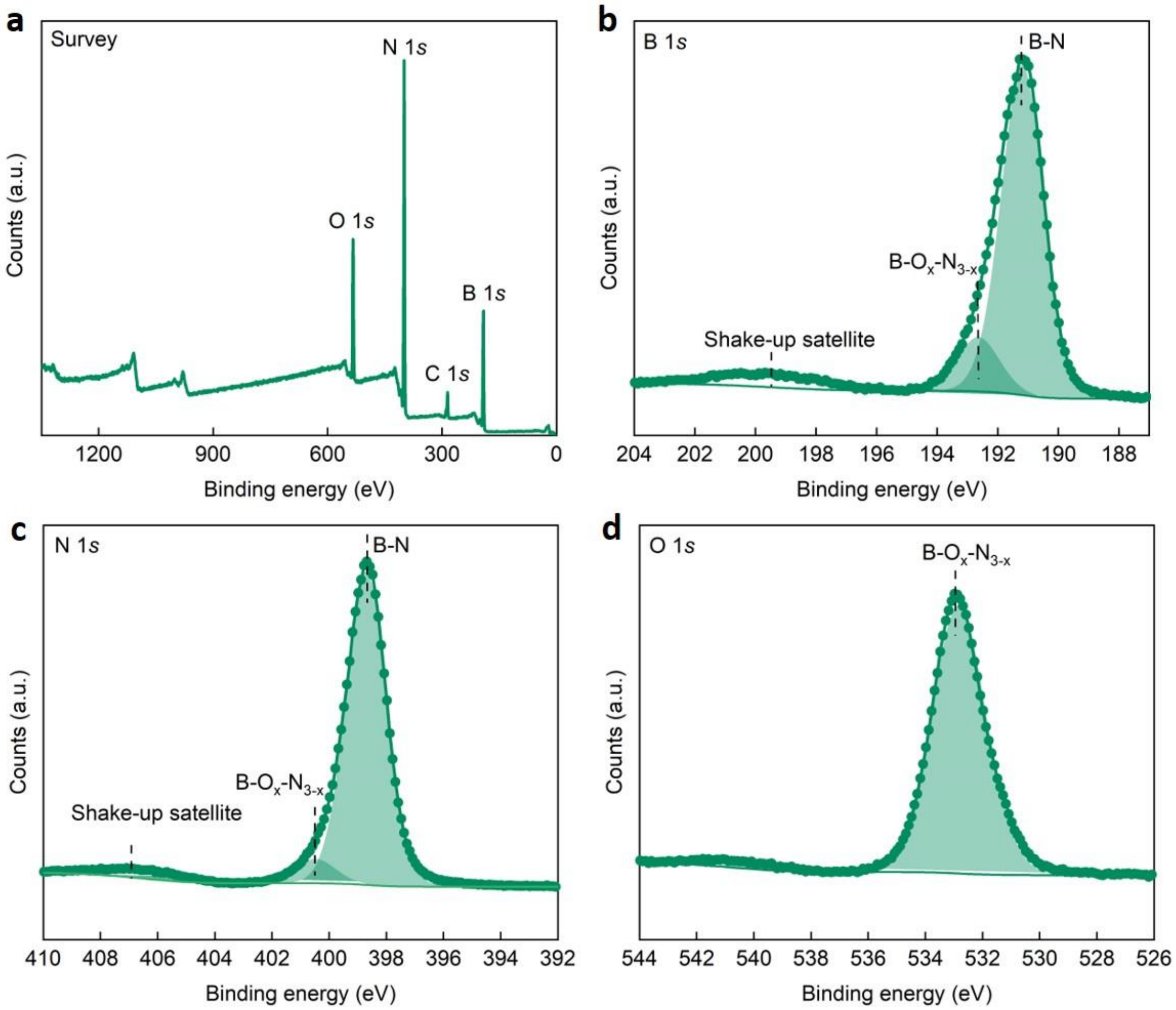

Fig. S6 I Chemical composition and states in m-BNO. Survey spectrum and high resolution XPS core level spectra for B 1*s*, N 1*s* and O 1*s* with the key characteristic peaks and corresponding binding energies highlighted. Synthesis parameters: 800 ^{o}C, 50 mL NH_3 min^{-1}, 2:1 molar ratio of metaboric acid to HMTA.

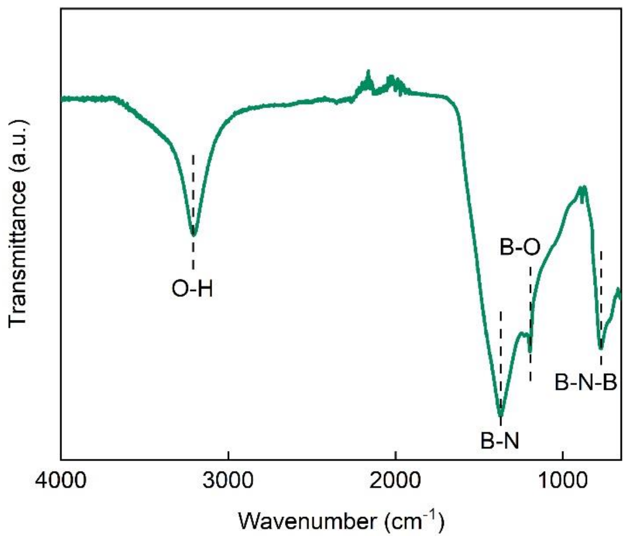

Fig. S7 I FT-IR spectrum for m-BNO**.**

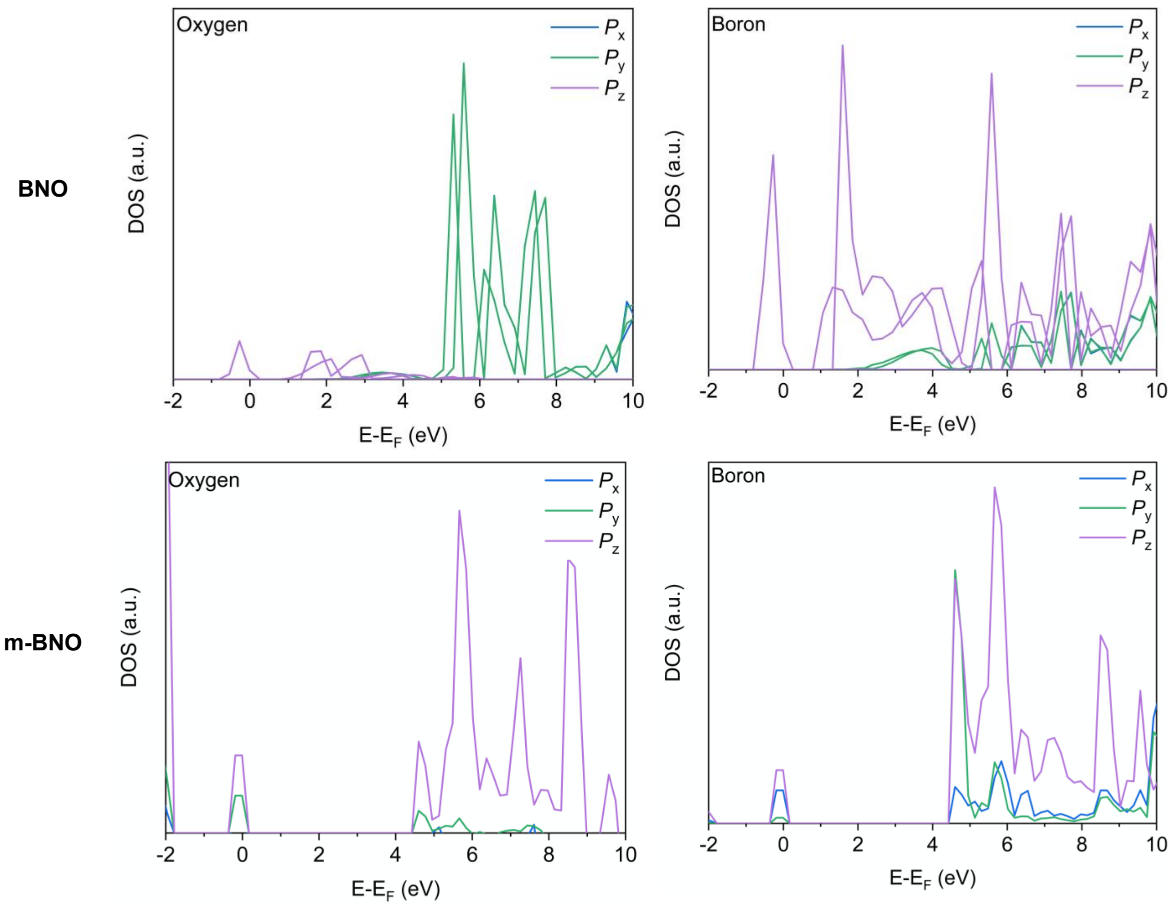

Fig. S8 I Orbital contributions from oxygen (left) and boron (right) atoms to PDOS in BNO sheet with all oxygen atoms as OB_3 states and m-BNO with all oxygen atoms as O-B-O states. The negative contributions have been “flipped” upwards.

References

[1] Shankar, R.; Mistry, E.; Lubert-Perquel, D.; Nevjestic, I.; Heutz, S.; Petit, C. A Response Surface Model to Predict and Experimentally Tune the Chemical, Magnetic and Optoelectronic Properties of Oxygen-Doped Boron Nitride. ChemRxiv 2021, doi.org/10.33774/chemrxiv-2021-zbgbj

[2] Bertoluzza, A., Monti, P., Battaglia, M. A. & Bonora, S. Infrared and raman spectra of orthorhombic, monoclinic and cubic metaboric acid and their relation to the “strength” of the hydrogen bond present. *J. Mol. Struct.* **64**, 123-136 (1980).
